# Mineral Deposition in Bacteria-Filled and Bacteria-Free Calcium Bodies in the Crustacean *Hyloniscus riparius* (Isopoda: Oniscidea)

**DOI:** 10.1371/journal.pone.0058968

**Published:** 2013-03-12

**Authors:** Miloš Vittori, Alenka Rozman, Jože Grdadolnik, Urban Novak, Jasna Štrus

**Affiliations:** 1 Department of Biology, Biotechnical Faculty, University of Ljubljana, Ljubljana, Slovenia; 2 National Institute of Chemistry, Ljubljana, Slovenia; 3 EN-FIST, Centre of Excellence, Ljubljana, Slovenia; INSERM U1059/LBTO, Université Jean Monnet, France

## Abstract

Crustacean calcium bodies are epithelial sacs which contain a mineralized matrix. The objectives of this study were to describe the microscopic anatomy of calcium bodies in the terrestrial isopod *Hyloniscus riparius* and to establish whether they undergo molt-related structural changes. We performed 3D reconstruction of the calcium bodies from paraffin sections and analyzed their structure with light and electron microscopy. In addition, we analyzed the chemical composition of their mineralized matrices with micro-Raman spectroscopy. Two pairs of these organs are present in *H. riparius*. One pair is filled with bacteria while the other pair is not. In non-molting animals, the bacteria-filled calcium bodies contain apatite crystals and the bacteria-free calcium bodies enclose CaCO_3_-containing concretions with little organic matrix. During preparation for molt, an additional matrix layer is deposited in both pairs of calcium bodies. In the bacteria-filled calcium bodies it contains a mixture of calcium carbonate and calcium phosphate, whereas only calcium carbonate is present in bacteria-free calcium bodies. After ecdysis, all mineral components in bacteria-free calcium bodies and the additional matrix layer in bacteria-filled calcium bodies are completely resorbed. During calcium resorption, the apical surface of the calcium body epithelium is deeply folded and electron dense granules are present in spaces between epithelial cells. Our results indicate that the presence of bacteria might be linked to calcium phosphate mineralization. Calcium bodies likely provide a source of calcium and potentially phosphate for the mineralization of the new cuticle after molt. Unlike other terrestrial isopods, *H. riparius* does not form sternal CaCO_3_ deposits and the bacteria-free calcium bodies might functionally replace them in this species.

## Introduction

The calcification of the rigid crustacean exoskeleton requires an abundant and readily available source of calcium ions after each molt. As a response, different groups of crustaceans have evolved various transient calcium deposits to retain some of the calcium necessary for cuticular calcification after molt [1], [2]. A general feature of terrestrial isopod crustaceans (Oniscidea) are sternal CaCO_3_ deposits, which form before ecdysis in the ecdysial space (the space between the detached old cuticle and the underlying epidermis) of anterior sternites [3]. The process of molt in isopods is biphasic. During the posterior ecdysis, animals shed the cuticle of body segments posterior to pereonite 4. In the anterior ecdysis that follows, the body segments anterior to pereonite 5 are molted. Sternal CaCO_3_ deposits are resorbed during the intramolt stage, the short period between the two partial ecdyses, and the mineral ions stored within them are utilized for the mineralization of the new exoskeleton [4].

Additionally, calcium deposits within specialized organs, the calcium bodies, have been reported in some representatives of the terrestrial isopod family Trichoniscidae [5], [6]. The microscopic anatomy of these organs and the changes they undergo during the molt cycle have thus far only been studied in the subterranean trichoniscid *Titanethes albus* [7]. In this species it was found that the calcium bodies are epithelial sacs filled with a bacteria-containing matrix mineralized with apatite. An additional glassy layer of mineralized matrix containing amorphous calcium minerals is formed in the calcium bodies of *T. albus* before molt and is degraded after it, suggesting that these organs have a role in calcium dynamics during molt. Whether the calcium bodies of other trichoniscids also harbor bacteria and what possible function these bacteria might perform is currently unknown.

In the trichoniscid *Hyloniscus riparius* (Koch), two pairs of calcium bodies, both located in pereonite 7, were described by Méhelÿ [6]. Later, Ziegler [8] reported a single pair of calcium-containing epithelial sacs in this species, with several separate mineral concretions present within each sac. Elemental analysis demonstrated large amounts of calcium in the concretions with phosphorus appearing in the posterior concretions only [8]. In this study, we determined the exact number, position and structure of calcium bodies using serial sectioning and 3D reconstruction of the organs. Contrary to the previous reports, our results demonstrate that two pairs of calcium bodies, each being a separate epithelial compartment, are present in *H. riparius*. Furthermore, we found that one pair of calcium bodies contains bacteria while the other pair does not.

Information on possible changes of the calcium body epithelium ultrastructure and the mineralization of calcium body matrix during the molt cycle in *H. riparius* is lacking. Also, the chemical composition of the material within the calcium bodies of *H. riparius* has not been determined so far. In order to establish whether or not changes occur in the mineralized matrix and the epithelium of calcium bodies during the molt cycle, we analyzed their structure in different molt cycle stages with a combination of microscopic techniques. In addition, we determined the composition of the mineralized matrices within these organs with micro-Raman spectroscopy.

Our results indicate that calcium bodies are involved in calcium dynamics during molt. In *H. riparius*, calcium bodies harboring bacteria as well as those lacking them are present within the same organism. The comparison of both types of calcium bodies can provide insights into which features of their epithelia and mineralized matrices are possibly linked to the presence of bacteria and it may indicate the possible role of bacteria within calcium bodies.

## Materials and Methods

### Animal Culture

Specimens of *H. riparius* were sampled near Cerknica and Ormož in Slovenia. Animals were kept in glass containers within a climate chamber at 21°C with a 14 h photoperiod. They were fed with mixed leaf litter from the sampling localities. For determination of molt cycle stages, individuals were separated into plastic containers with wet filter paper and observed daily. Specimens in the 2–4 mm body length range were used in our analyses. No specific permits were required for field sampling.

### Examined Stages of the Molt Cycle

We examined animals in five different stages of the molt cycle. Specimens with no sign of apolysis and in which ecdysis was not previously observed were considered to be in the intermolt stage. During this stage the exoskeleton is fully formed and the integumental epithelium does not synthesize cuticle. The absence of new cuticle synthesis was chosen as the criterion for intermolt stage. To rule out possible confusion with premolt stage specimens, the lack of new cuticle synthesis was confirmed microscopically on tissue sections of the posterior body-half in all specimens examined.

The premolt stage is the period from the apolysis (the detachment of the old cuticle from the epidermis) to the beginning of ecdysis [9] and in most terrestrial isopods the onset of premolt stage is easily recognized by the formation of sternal CaCO_3_ deposits [10]. Since we established that sternal deposits are not formed in *H. riparius* (see Results), we identified premolt specimens by the presence of a transparent posterior edge on the pereon epimeres. In all such specimens, we additionally confirmed the presence of an ecdysial gap and the synthesis of the new cuticle, which were chosen as criteria for the premolt stage, on tissue sections of the posterior body-half.

Individuals in intramolt stage (between the posterior and anterior ecdyses) and postmolt stage (after the anterior ecdysis) were recognized by direct observation of the posterior ecdysis and the anterior ecdysis, respectively. Specimens were studied at two points in the postmolt stage: within a day after anterior ecdysis and two days after anterior ecdysis.

### Histology

For serial paraffin sections and 3D reconstruction of calcium bodies, *H. riparius* specimens were anesthetized with diethyl ether and fixed overnight in 4% aqueous formaldehyde at room temperature. The fixative was then washed and whole specimens were decalcified in 10% ethylenediaminetetraacetic acid (EDTA), dehydrated in an ethanol series and embedded in paraffin. Tissue sections (8 µm) were placed on glass slides, deparaffinized with xylene, rehydrated in an ethanol series, stained with hematoxylin and eosin and covered in Pertex (Medite). The shape of the calcium bodies’ outer surface was reconstructed in the program Reconstruct [11] by manually tracing the calcium body epithelium on images of sequential sections. For examination of the mineralized matrices within calcium bodies in different molt cycle stages, whole specimens were fixed in 96% ethanol to prevent the dissolution of mineral components. Sections were prepared as described above and stained with eosin. Light micrographs were captured on an AxioImager Z.1 microscope (Zeiss) with the HRc Axiocam camera.

### Semithin Sections and Transmission Electron Microscopy (TEM)

For transmission electron microscopy, pereons of *H. riparius* in different molt cycle stages were fixed in 2.5% glutaraldehyde and 2% paraformaldehyde in 0.1 M cacodylate buffer, pH = 7.3 at 4°C for several days. After washing of the fixative with 0.1 M cacodylate buffer, tissues were postfixed with 1% aqueous OsO_4_ for 1 h, dehydrated in an ethanol series and embedded in Spurr’s resin (SPI). Semithin sections (0.5 µm) were transferred to glass slides, stained with Azur II and Methylene blue [12] and imaged as described for the paraffin sections. Thin sections (70 nm) were collected on formvar-coated copper grids, contrasted with uranyl acetate and lead citrate and observed with a CM 100 transmission electron microscope (FEI). The preparation procedures for TEM generally resulted in the loss of calcium minerals from tissues.

### Scanning Electron Microscopy (SEM)

For scanning electron microscopy, isolated calcium bodies or pereon halves were immersed in methanol for several hours and air dried in order to preserve mineral components. Alternatively, pereon halves of some specimens were fixed as for TEM, postfixed with 1% OsO_4_ for 2h, dehydrated in an ethanol series followed by two changes of pure acetone and air-dried in Hexamethyldisilazane (HMDS) for the preservation of soft tissues. Dry samples were attached to brass holders with silver paint and fractured to expose the interior. Specimens were coated with a 14 nm thick layer of platinum using a BAL-TEC SCD 050 sputter coater and observed with a JEOL JSM-7500F field-emission scanning electron microscope.

### Micro-Raman Spectroscopy

For micro-Raman spectroscopy, specimens were decapitated and dissected in absolute ethanol or methanol. Individual calcium bodies were isolated, air dried, placed on glass slides and covered with cover slips. For comparison, sternal deposits of *Titanethes albus* (Koch) were prepared the same way.

Raman spectra were recorded on a Bruker Ramanscope III by application of laser excitation at 1064 nm. The laser radiation was transmitted to the microscope with an optical fiber and focused on the sample with a long working distance objective at ×40 magnification. The Raman scattered light was collected with the same objective and detected with a liquid-nitrogen cooled Ge detector. Spectra were averaged from ∼19.000 interferograms and apodised with a Happ-Henzel function. All spectra were recorded in the spectral range between 50 and 3600 cm^−1^ with the nominal resolution of 4 cm^−1^. Laser power at the sample was estimated to be approximately 100 mW. Reducing the laser power did not result in significant changes in the Raman spectra.

## Results

### 1. Molting in *H. riparius*

**Figure 1:**
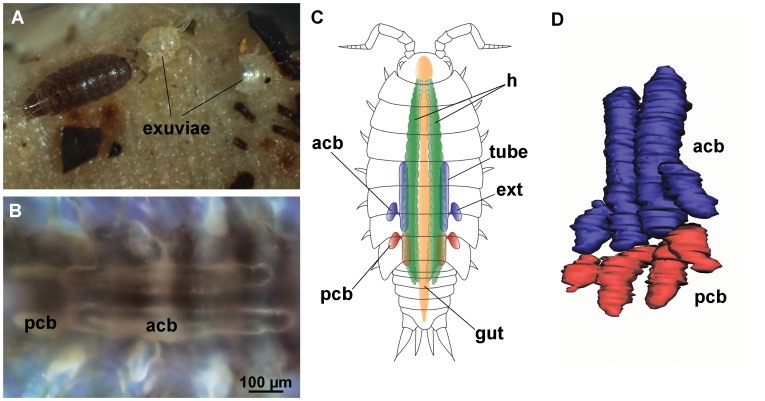
Molting and calcium bodies in *Hyloniscus riparius*. (A) An individual shortly after anterior ecdysis. Exuviae of both body-halves are visible in the proximity. (B) Dark-field image of the posterior (pcb) and anterior (acb) calcium bodies *in situ*. (C) An illustration of the position of the digestive system and the calcium bodies in the body. Each calcium body consists of a medial tube and a lateral extension (ext). (D) 3D reconstruction of calcium bodies from serial paraffin sections, dorsolateral view. h – hepatopancreas.

As in other isopods, the molt in *H. riparius* is biphasic (Fig. 1 A) with the anterior ecdysis following the posterior ecdysis in less than a day. Animals that we observed never consumed the posterior exuviae, but often consumed the anterior exuviae after the anterior ecdysis.

Although animals in all molt cycle stages were carefully examined, no sternal deposits were ever observed in premolt and intramolt specimens (Fig. S1). Furthermore, the anterior sternal epithelium in intramolt stage lacks the ultrastructural characteristics reported in other terrestrial isopods during the resorption of sternal deposits (Fig. S1), such as a basolateral intercellular network and invaginations of the apical plasma membrane [13]. Thus, sternal CaCO_3_ deposits are apparently entirely absent in *H. riparius*.

### 2. Number, Position and Structure of Calcium Bodies

As demonstrated by serial sectioning and 3D reconstruction from paraffin sections, two pairs of calcium bodies are present in the pereon of *H. riparius* (Figs. 1 B–D). The larger anterior pair extends from pereonite 4 to the middle of pereonite 6 and the smaller posterior pair from pereonite 6 to pleonite 1. Within pereonite 6, both pairs overlap as the anterior calcium bodies extend dorsally over the posterior ones; however, we established that there is no luminal connection between the anterior and posterior calcium bodies. Each calcium body is an epithelial sac consisting of a longitudinally oriented medial tube with a smaller lateral extension (Fig. 1 B). The organs are positioned ventrally in the body, close to the hindgut and the hepatopancreatic tubes and the basal laminae of anterior calcium bodies and hepatopancreas are closely apposed. The anterior and posterior calcium bodies differ markedly in their contents; therefore, each pair will be considered separately.

**Figure 2:**
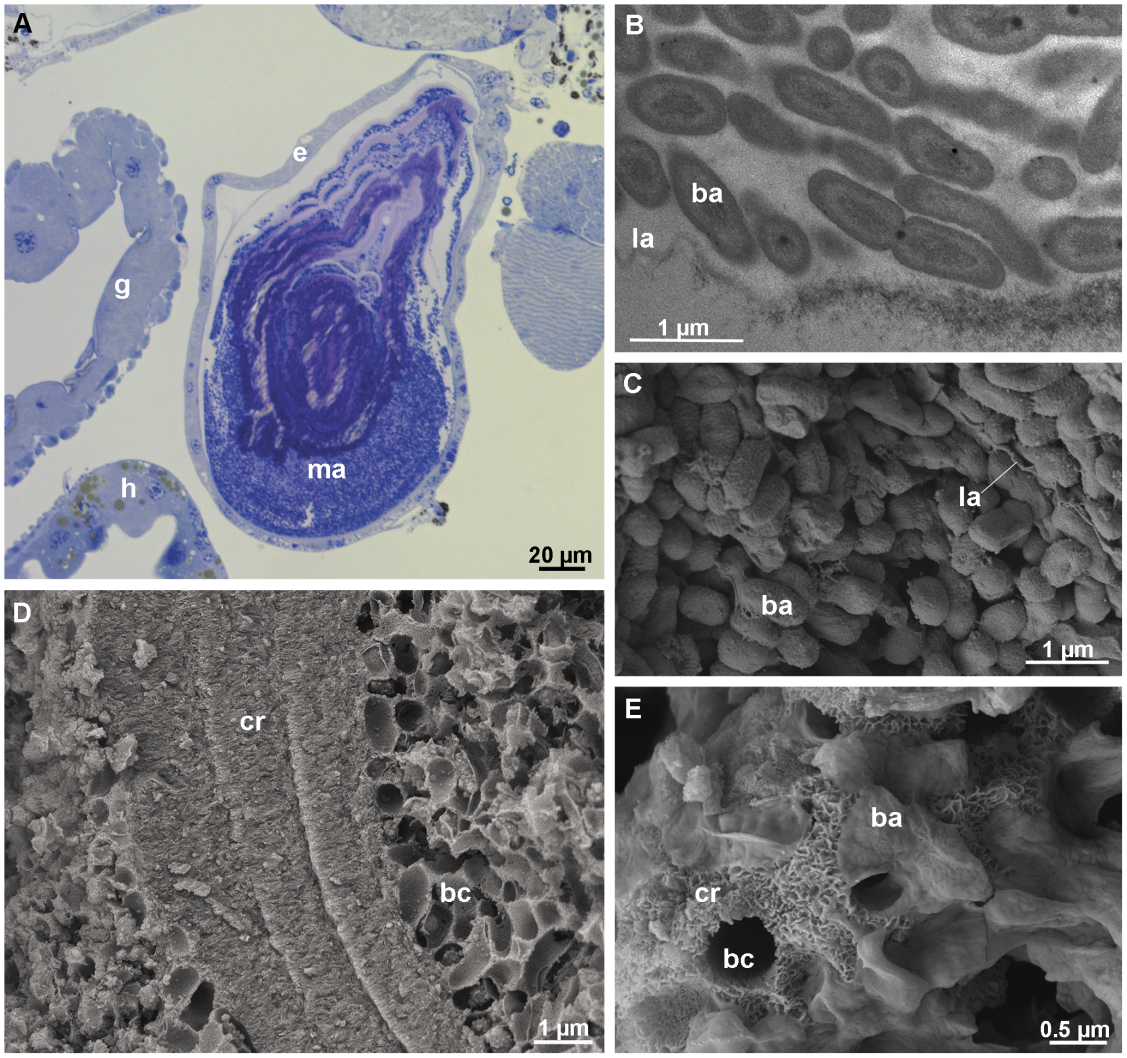
Structure and contents of the posterior calcium bodies in intermolt stage. (A) Semithin section through a posterior calcium body. A single-layered epithelium (e) encloses the layered, bacteria-containing matrix (ma). (B) Transmission electron micrograph showing bacteria (ba) in the proximity of a lamina (la) within a posterior calcium body. (C) Scanning electron micrograph (aldehyde/OsO_4_ fixation) of the bacteria within the posterior calcium body. An extracellular lamina (la) is visible among the bacteria (D) Scanning electron micrograph of the matrix within the posterior calcium bodies (methanol fixation). Layers of crystals (cr) and numerous bacterial casts (bc) are visible. (E) Scanning electron micrograph (methanol fixation) of crystals (cr) within the posterior calcium bodies. g – gut, h – hepatopancreas.

Each posterior calcium body consists of a single-layered epithelium enclosing layered material (Fig. 2 A). As is evident from TEM and SEM observations, the posterior calcium bodies harbor large numbers of rod-shaped bacteria approximately 0.8 µm in length and 0.4 µm in diameter (Figs. 2 B, C). Dense layers of crystals approximately 100 nm in size are present among the bacteria (Figs. 2 D, E). Crystals are often associated with thin laminae, resulting in a layered appearance of the mineralized matrix (Fig. 2 D).

**Figure 3:**
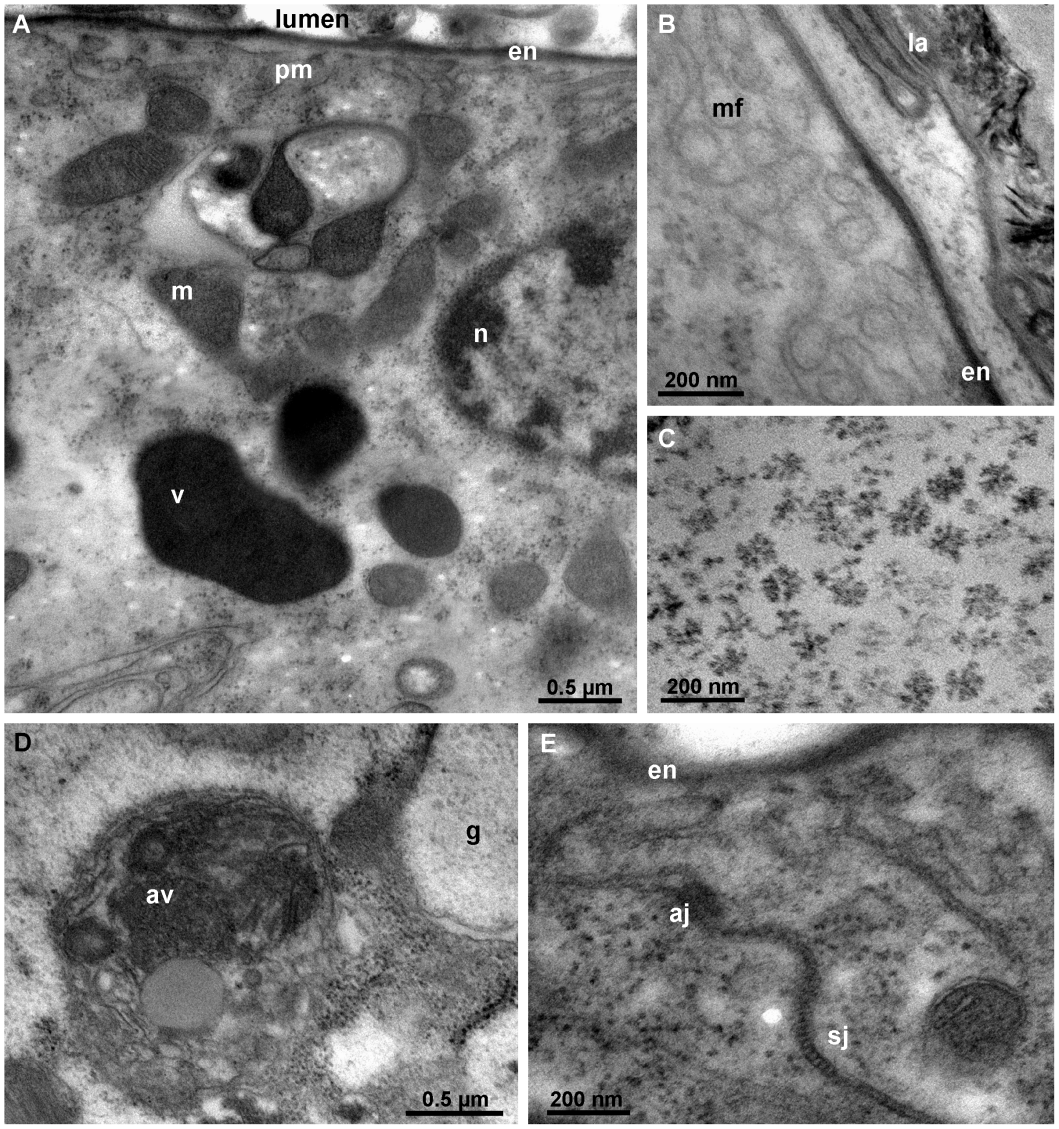
Ultrastructure of the posterior calcium body epithelium in intermolt stage. (A) Section through the epithelium (apical side up). An extracellular envelope (en) lines the folded apical plasma membrane (pm). Mitochondria (m) and electron dense vesicles (v) are present in the cytoplasm. (B) The apical surface of the posterior calcium body epithelium. The apical plasma membrane forms folds (mf). The envelope (en) consists of a proximal electron dense and a distal electron lucent layer. A lamina (la) is visible inside the lumen. (C) Rosettes of electron dense particles (most likely glycogen) in the cytoplasm of the epithelial cells. (D) An autophagic vacuole (av) in the cytoplasm of an epithelial cell. (E) Junction between two epithelial cells. The subapical adherens junction (aj) and the more basally positioned septate junction (sj) are visible. g – glycogen, n – nucleus.

The posterior calcium body epithelium is lined apically by a 45 nm thick extracellular envelope, which encloses the material in the lumen (Fig. 3 A). The envelope consists of a thinner electron lucent layer facing the lumen and a thicker electron dense layer in contact with the epithelium (Fig. 3 B). Additional thin laminae are often present in the central area of the calcium body lumen. The cytoplasm of epithelial cells with intensely folded apical membranes contains numerous mitochondria and abundant electron dense particles, which form rosettes and structurally resemble glycogen (Fig. 3 C). In addition, electron dense vesicles (Fig. 3 B) and large organelles filled with membranous structures (possibly autophagic vacuoles) are common in the cytoplasm of epithelial cells (Fig. 3 D). Cells interconnect by subapical adherens junctions and by more basally positioned septate junctions (Fig. 3 E).

**Figure 4:**
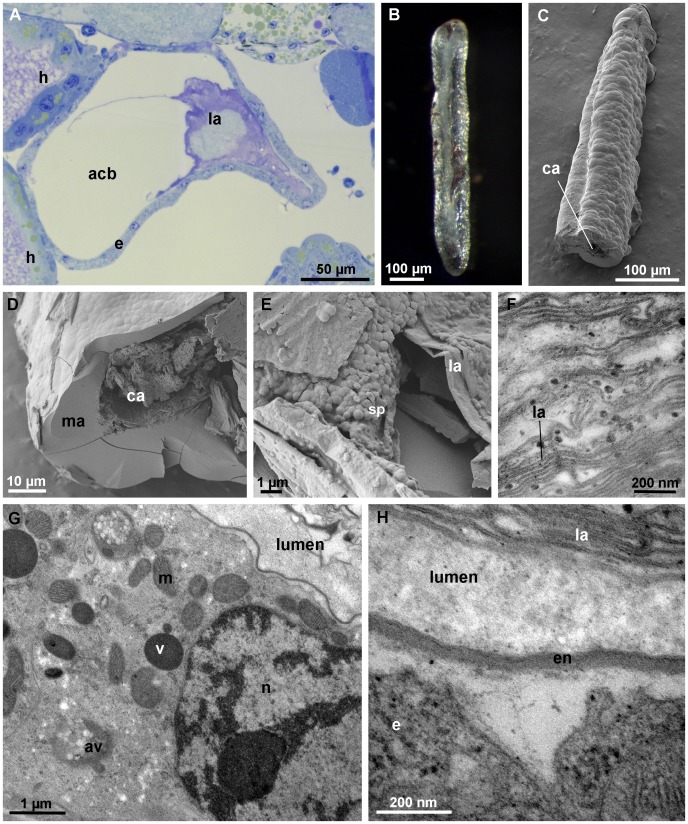
Anterior calcium bodies in intermolt stage. (A) Semithin section through an anterior calcium body (acb). An epithelium (e) forms the wall of the organ. An agglomeration of laminae (la) is visible in the lumen. Mineral content was lost during tissue preparation. (B) Mineral concretion from the medial tube of an anterior calcium body. (C) Scanning electron micrograph of a fractured mineral concretion from an anterior calcium body (methanol fixation) with a central cavity (ca). (D) Scanning electron micrograph of a fractured anterior calcium body (methanol fixation) showing a continuous layer of mineralized matrix (ma) formed around the central cavity (ca) with folded laminae. (E) Higher magnification scanning electron micrograph showing small spherules (sp) on the surface of laminae (la) within the central cavity. (F) Transmission electron micrograph of the laminae (la) within an anterior calcium body. (G) Cross-section through the anterior calcium body epithelium. The cytoplasm of epithelial cells contains numerous mitochondria (m), electron dense vesicles (v) and autophagic vacuoles (av). The apical plasma membrane is not folded. (H) The apical surface of an epithelial cell (e). The apical plasma membrane is lined by an envelope (en) consisting of a proximal electron dense and a distal electron lucent layer. Numerous laminae (la) are visible in the lumen. h – hepatopancreas, n- nucleus.

As in posterior calcium bodies, the wall of the anterior calcium bodies is formed by a single-layered epithelium (Fig. 4 A), but no bacteria were ever observed in their lumen. Instead, the anterior calcium bodies in intermolt stage contain glassy concretions (Figs. 4 B, C). These concretions consist of a continuous layer of mineralized matrix that encloses a central cavity (Figs. 4 C, D). This cavity contains numerous plied laminae, the surfaces of which are covered with small spherules less than 1 µm in size (Fig. 4 E). A lamina also lines the inner surface of the continuous mineralized matrix. As visible with TEM, the laminae are 10 nm thick and electron dense particles are present among them. The spherules visible with SEM are not preserved during specimen preparation for TEM.

The anterior calcium body epithelium in the intermolt stage is ultrastructurally similar to the posterior calcium body epithelium in most respects. Numerous mitochondria and electron dense particles are present in the anterior calcium body epithelium (Fig. 4 G), and its apical side is lined by an envelope very similar to the envelope in the posterior calcium bodies (Fig. 4 H). A notable difference between the two pairs of organs is that the apical plasma membrane of the anterior calcium body epithelium is not folded in the intermolt stage (Figs. 4 G, H).

### 3. Structural Dynamics of Calcium Bodies during the Molt Cycle

#### Premolt stage

**Figure 5:**
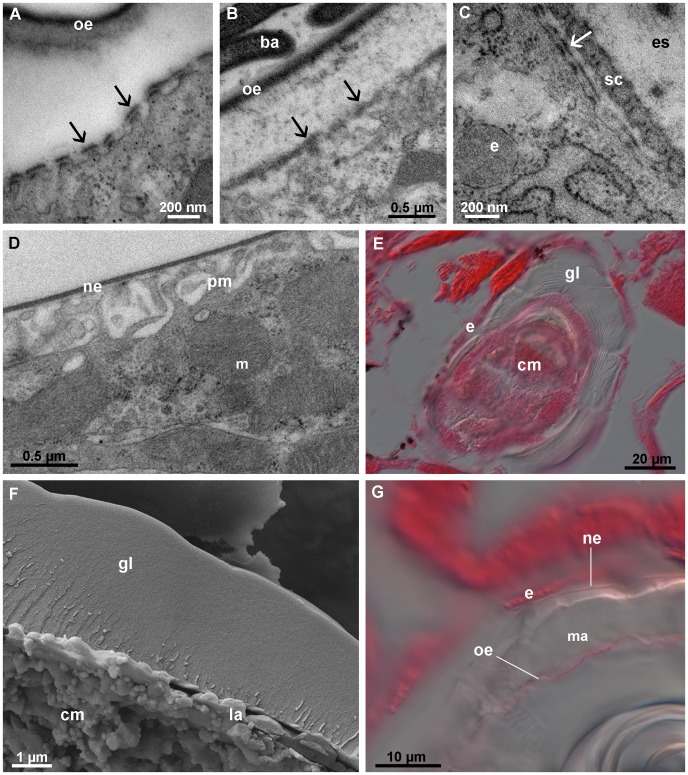
Calcium bodies in premolt stage. (A) Synthesis of the new envelope (arrows) in an anterior calcium body. The new envelope is discontinuous and is deposited over short protrusions of the apical plasma membrane. The old envelope (oe) is detached. (B) Synthesis of the new envelope (arrows) in the posterior calcium bodies. (C) Synthesis of the tergal epicuticle (arrow) in early premolt stage. A forming cuticular scale (sc) is visible. (D) The apical surface of the anterior calcium body epithelium in late premolt stage. The folded apical plasma membrane (pm) is lined by the new envelope (ne). (E) Differential interference contrast image of a posterior calcium body in late premolt stage (eosin stain). A glassy layer (gl) of mineralized matrix is present between the bacteria-containing central matrix (cm) and the epithelium (e). (F) Scanning electron micrograph of the glassy layer (gl) in a fractured posterior calcium body (methanol fixation). Small spherules are visible on the laminae (la) in the outer parts of the central matrix. (G) Differential interference contrast image of a premolt stage anterior calcium body (eosin stain). The old envelope (oe) is embedded in the mineralized matrix (ma). The new envelope (ne) lines the epithelium (e). ba – bacterium, es – ecdysial space, h – hepatopancreas, m – mitochondrion.

In early premolt stage, as the epidermis is synthesizing the new epicuticle, a new envelope is secreted in both pairs of calcium bodies (Figs. 5 A, B). The new envelope is formed over dense plaques on short protrusions of the plasma membrane and is initially discontinuous (Fig. 5 A). In this respect, the process of deposition of the new envelope within the calcium bodies is ultrastructurally similar to the deposition of the new integumental epicuticle, which is secreted at the same time (Fig. 5 C). During the premolt stage, the apical plasma membrane is folded in the anterior calcium bodies as well (Fig. 5 D). In other respects, the ultrastructure of the premolt stage calcium body epithelia is similar to that of epithelia in the intermolt stage.

After the new envelope is synthesized, additional mineralized matrix is formed in the space between the detached old envelope and the new envelope in both pairs of calcium bodies. In the posterior calcium bodies, a glassy layer of mineralized matrix develops in the space between the old envelope and the new one (Figs. 5 E, F). Small spherules may also form along the laminae positioned central to the old envelope (Fig. 5 F). In the anterior calcium bodies, the old envelope appears to be incorporated into the forming mineralized matrix (Fig. 5 G), which resembles the matrix present within the lumen in intermolt stage.

#### Intramolt stage

**Figure 6:**
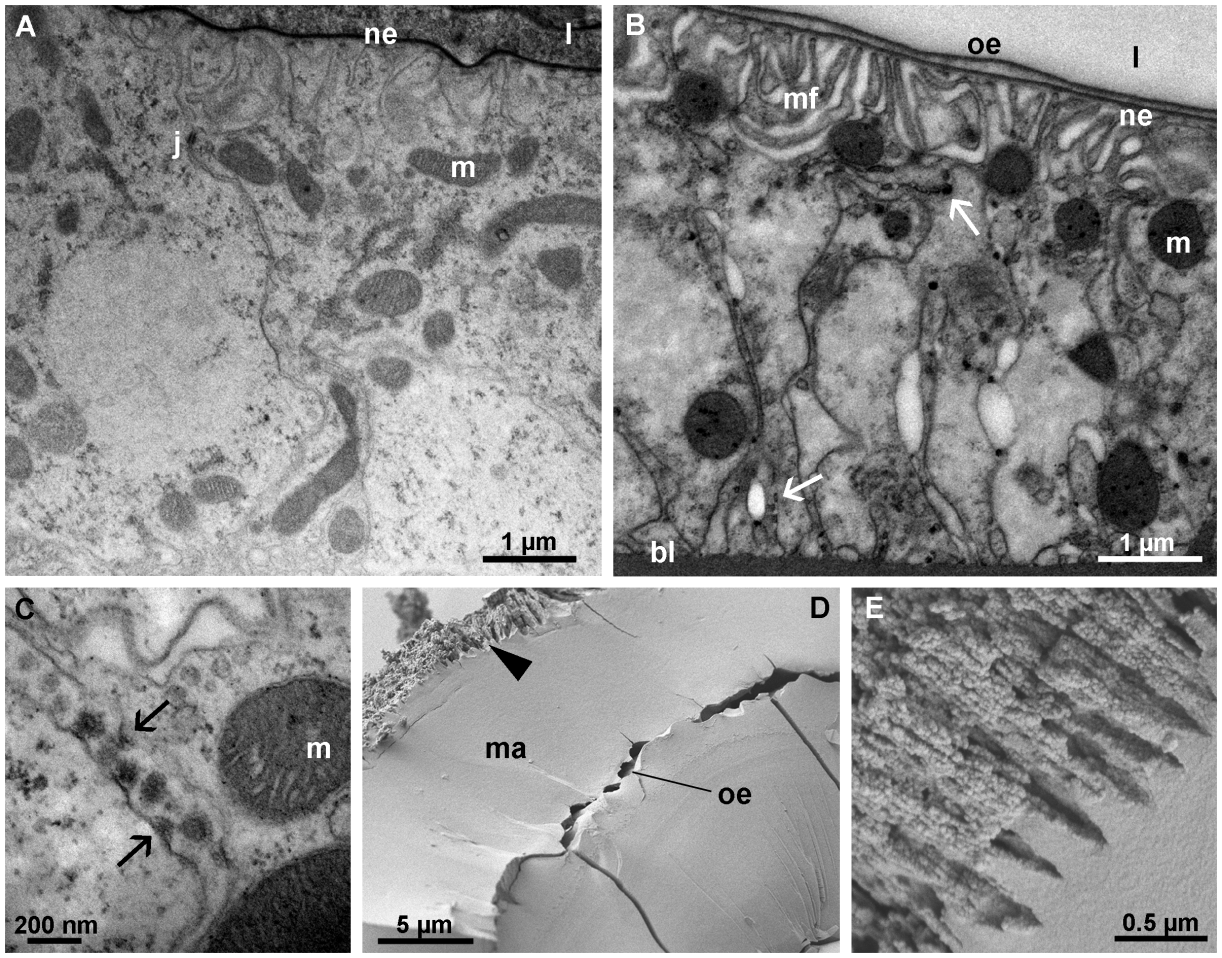
Calcium bodies in intramolt stage. (A) The posterior calcium body epithelium is unaltered with respect to the intermolt stage. (B) The anterior calcium body epithelium forms deep apical membrane folds (mf). Electron dense granules (arrows) are present in intercellular spaces between neighboring cells. Mineral components were lost during tissue preparation. (C) Formation of electron dense granules in intercellular spaces of the anterior calcium body epithelium on short protrusions (arrows) of the plasma membrane. (D) The mineralized matrix (ma) in an anterior calcium body. The outer surface of the mineral concretion (upper left side of image) appears etched (arrowhead). The embedded old envelope (oe) separates two layers of the mineralized matrix (ma). (E) Higher magnification of the etched surface of the mineralized matrix within an anterior calcium body showing granular material arranged in radial threads. The surface of the concretion is to the upper left side of the image. bl – basal lamina, j – cell junction, l – calcium body lumen, m- mitochondrion, ne – new envelope, oe – old envelope.

The ultrastructure of the posterior calcium body epithelium in intramolt stage specimens appears unaltered with respect to the premolt stage epithelium (Fig. 6 A). By contrast, the apical epithelial surface in the anterior calcium bodies in intramolt stage is greatly enlarged by the formation of numerous membrane folds (Fig. 6 B). In addition, electron dense granules 50–100 nm in diameter are present in the intercellular spaces between adjacent epithelial cells (Fig. 6 B). These granules are formed on short protrusions of the lateral plasma membrane (Fig. 6 C) and are present basally to the subapical cell junctions.

The material within calcium bodies in intramolt stage is similar to that found in late premolt stage, i.e. the matrix of the anterior calcium bodies is extensive (Fig. 6 D) and the glassy layer in the posterior calcium bodies is well developed (not shown). However, the outer surface of the mineralized matrix formed during the premolt stage in anterior calcium bodies appears etched, which suggests that the matrix is being dissolved in intramolt stage. Radially oriented columns of small granules (about 30 nm in diameter) are visible on the etched surface (Figs. 6 D, E).

#### Postmolt stage

**Figure 7:**
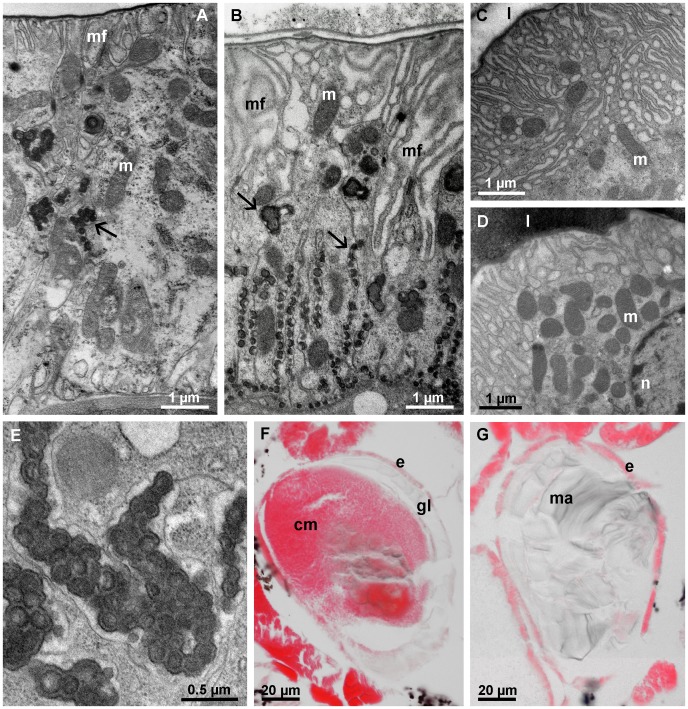
Calcium bodies one day after anterior ecdysis. (A) The posterior calcium body epithelium (apical side up) with apical membrane folds (mf) and electron dense granules (arrow) in intercellular spaces between neighboring cells. (B) The anterior calcium body epithelium (apical side up). Folds (mf) of the apical plasma membrane reach deeply into the epithelium. Electron dense granules of varying sizes (arrows) are present in intercellular spaces. (C) Oblique section through the apical part of the anterior calcium body epithelium showing numerous folds of the apical plasma membrane. (D) Oblique section through the apical part of the posterior calcium body epithelium with numerous membrane folds and mitochondria (m). (E) High magnification image of the electron dense granules in intercellular spaces of the anterior calcium body epithelium. (F) Paraffin section of the posterior calcium body (eosin stain) showing the glassy layer (gl), which is still present after anterior ecdysis. (G) Paraffin section of the anterior calcium body (eosin stain) showing the presence of mineralized matrix (ma) within it. cm – central matrix, e – epithelium, l – lumen, n – nucleus.

During the first day following anterior ecdysis, the epithelial cells in both pairs of calcium bodies have a deeply folded apical membrane, electron dense granules in the basolateral parts of the spaces between adjacent cells are numerous (Figs. 7 A–E). At this time, the glassy layer is still present within the posterior calcium bodies and the anterior calcium bodies contain variable amounts of mineralized matrix (Figs. 7 F, G).

**Figure 8:**
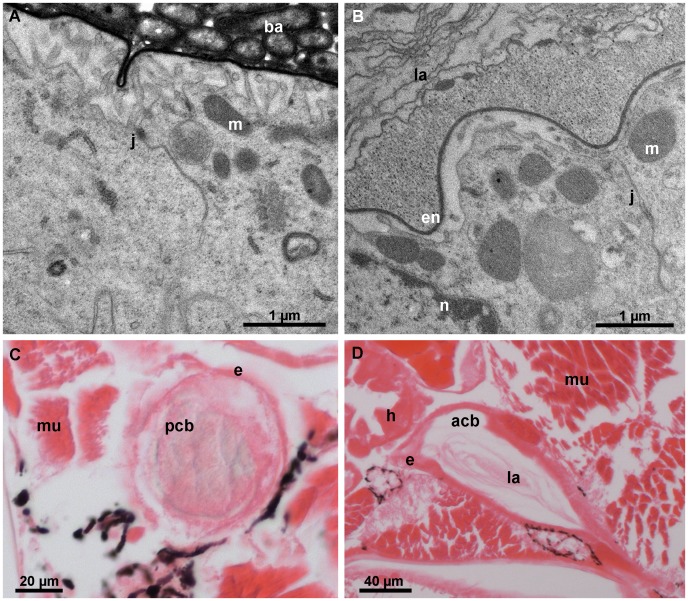
Calcium bodies two days after anterior ecdysis. (A) The posterior calcium body epithelium. Epithelial ultrastructure is similar to that of the intermolt stage epithelium. (B) The anterior calcium body epithelium. The apical surface of epithelial cells is no longer folded and adjacent cells are in close contact. (C) Paraffin section (eosin stain) of a posterior calcium body (pcb). No glassy layer is present. (D) Paraffin section (eosin stain) of an anterior calcium body (acb). The lumen is devoid of mineral and only organic laminae (la) are visible within it. ba – bacteria, e – epithelium, en – envelope, h – hepatopancreas, j – cell junction, m – mitochondrion, mu – muscle, n – nucleus.

Two days after the anterior ecdysis, the ultrastructure of the calcium body epithelium is restored to its intermolt stage condition (Figs. 8 A, B). Electron dense granules are absent from the intercellular spaces between epithelial cells and adjacent plasma membranes are in close apposition. In anterior calcium bodies, the apical plasma membrane is smooth and lacks invaginations, whereas in the posterior calcium bodies, the apical membrane folds are still present. The posterior calcium bodies lack a glassy layer at this stage; however, the bacteria and crystals remain in the central matrix (Fig. 8 C). The anterior calcium bodies, on the other hand, are completely devoid of the mineralized matrix and only numerous laminae remain in the lumen (Fig. 8 D), showing that the mineralized matrix in anterior calcium bodies is completely degraded during the two days following exuviation.

### 4. Micro-Raman Spectroscopy

**Figure 9:**
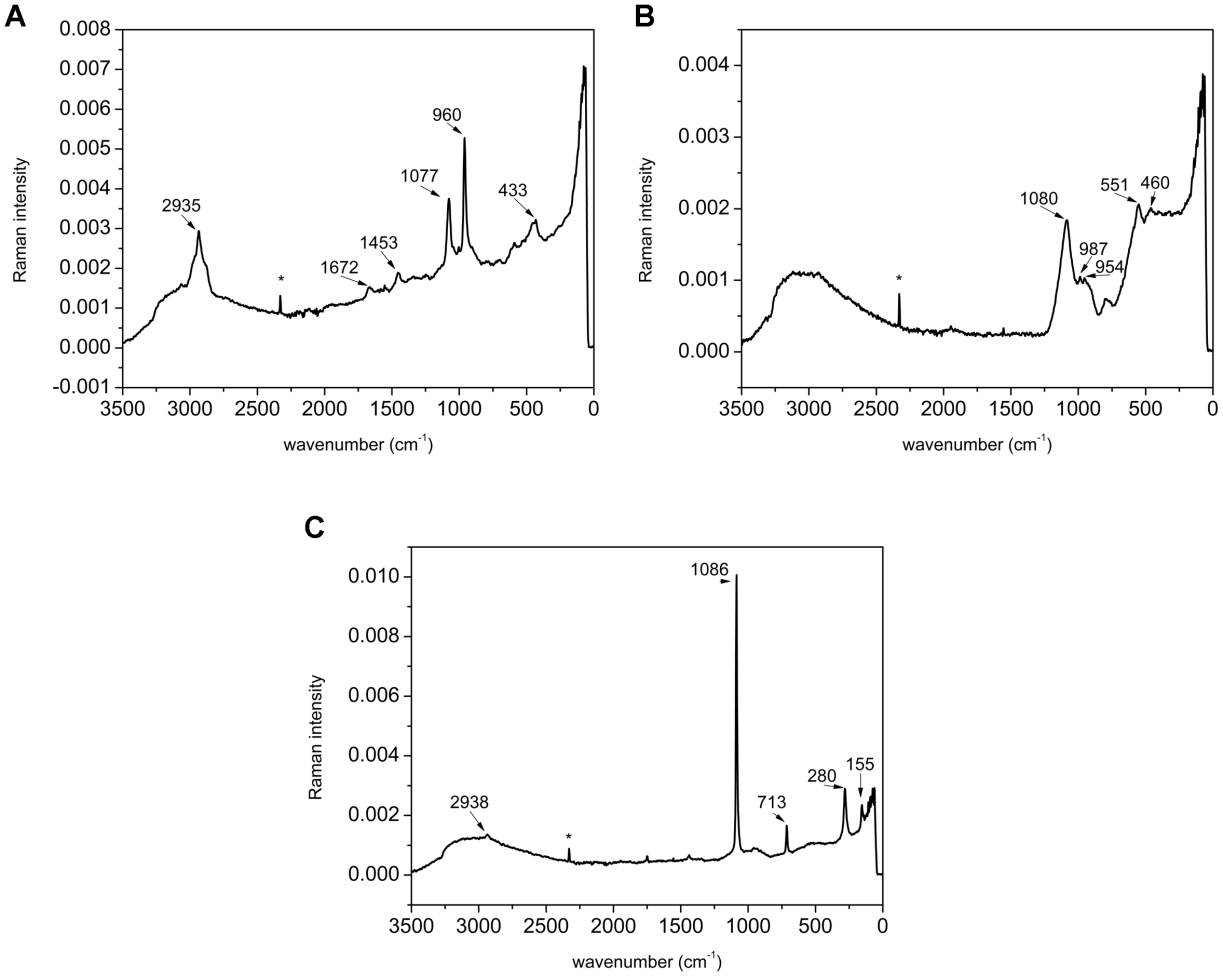
Raman spectra of mineralized matrices within calcium bodies. (A) Raman spectrum of the bacteria-containing central matrix in a posterior calcium body. (B) Raman spectrum of the glassy layer developed during the premolt stage in a posterior calcium body. (C) Raman spectrum of the concretion within an anterior calcium body. Positions of the most prominent peaks are given in cm^−1^. Asterisks denote spikes.

The Raman spectrum of the bacteria-containing central matrix within posterior calcium bodies (Fig. 9 A) demonstrates the presence of carbonated apatite [14], [15]. The prominent peak at 960 cm^−1^ corresponds to the ν_1_ stretching of PO_4_ [14], [16] and the peak at 433 to the ν_2_ stretching of PO_4_ in apatite [15]. The peak at 1077 cm^−1^ is contributed by the ν_1_ stretching of CO_3_ [14]. The demonstration of carbonated apatite is in accordance with the finding of crystals within the central matrix using SEM. At high frequencies the most prominent peak is located at 2935 cm^−1^. This peak is associated with CH stretching and the peak at 1453 cm^−1^ with CH_2_ wagging vibrations [17]. These peaks demonstrate the presence of large quantities of organic substances within the central matrix of posterior calcium bodies and can be attributed either to the abundant bacteria within the matrix [18] or to organic matrix components. The broad peak from 3000 to 3300 cm^−1^ corresponds to OH stretching of bound water [19].

The spectrum of the glassy layer, which develops in the posterior calcium bodies during premolt stage, is characterized by three prominent peaks at 954 cm^−1^, 987 cm^−1^ and 1080 cm^−1^ (Fig. 9 B). The peak at 954 cm^−1^, attributed to ν_1_ stretching of PO_4_, is characteristic of amorphous calcium phosphate (ACP). Unlike apatite, which features a narrow peak at about 960 cm^−1^, the corresponding peak in ACP is broader and shifted towards smaller wavenumbers [15], [16]. An additional peak of similar intensity at 987 cm^−1^ matches the ν_1_ stretching of PO_4_ in calcium hydrogen phosphates, i.e., brushite [20] and monetite [21]. However, the obtained spectrum does not allow us to draw firm conclusions about the presence of these minerals in the samples. If calcium hydrogen phosphates contribute to this peak, the presence of brushite is more likely, since its dehydration to monetite generally requires harsh conditions [22]. The peak at 1080 cm^−1^ is the strongest in the spectrum and, considering the broadness of this peak, is contributed by amorphous calcium carbonate [19]. The complexity of the spectrum in the 200–900 cm^−1^ region likely results from several mineral phases occurring together. Unlike the central matrix, the glassy layer of the posterior calcium bodies contains relatively small amounts of organic matrix components and consists mostly of minerals.

The Raman spectrum obtained from the mineralized matrix within the anterior calcium bodies (Fig. 9 C) demonstrates the presence of calcite, with peaks at 1086 cm^−1^ (symmetric ν_1_ stretching of CO_3_) [23], 713 cm^−1^ [19], 155 cm^−1^ and 280 cm^−1^ (calcite lattice vibration) [24]. The characteristic peaks at 155 cm^−1^ and 280 cm^−1^ distinguish calcite from amorphous calcium carbonate (ACC) [19] as well as vaterite and aragonite [25]. The spectrum lacks characteristic calcium phosphate peaks and peaks attributable to organic components are very small. Similar spectra were obtained from specimens in intermolt and intramolt stages. Analysis of the mineralized matrix in samples isolated in absolute ethanol and methanol gave identical results.

## Discussion

The results of this study show that bacteria are present in calcium bodies of trichoniscids other than *T. albus*, in which they were first reported [7], and are likely a general feature of calcium bodies. However, unlike *T. albus*, in which both pairs of calcium bodies harbor bacteria and are similar in structure and composition, only the posterior pair of calcium bodies contains bacteria in *H. riparius*, whereas the anterior pair is devoid of them.

There are two notable differences between the anterior and posterior calcium bodies of *H. riparius*. Firstly, the apical plasma membrane of the epithelium is deeply folded throughout the molt cycle in the posterior calcium bodies, whereas in the anterior pair it is smooth in intermolt stage and is deeply folded only during intramolt and postmolt stages and to a lesser extent during premolt stage, which may be linked to ion transport and matrix secretion. Secondly, the posterior calcium bodies continuously contain apatite and the transient glassy layer within them contains a high amount of calcium phosphate, whereas the mineralized matrix of the anterior calcium bodies contains only CaCO_3_-containing transient deposits. Our results are in agreement with the elemental analysis of calcium bodies in non-molting *H. riparius* [8], which demonstrated the presence of large amounts of phosphorus in the posterior mineral concretions, whereas no phosphorus was detected in the anterior ones.

On the basis of our results we propose the following model of mineral deposition and resorption in the calcium bodies of *H. riparius*.

Before apolysis, deposition of a CaCO_3_-containing mineralized matrix begins around the laminae in the lumen of anterior calcium bodies. In the posterior calcium bodies, only the bacteria-containing matrix mineralized with apatite is present at this time. Later, the old envelopes detach from the calcium body epithelia and new envelopes are synthesized. These events are concomitant with integumental apolysis and the synthesis of the new epicuticle. Later in premolt stage, additional mineralized matrix is formed between the old envelope and the new envelope in both pairs of calcium bodies. In the anterior calcium bodies, the mineralized matrix contains only CaCO_3_, whereas in the posterior calcium bodies, the newly formed glassy layer contains a mixture of calcium carbonate and calcium phosphate, mostly in amorphous form. After the posterior ecdysis, degradation of the mineralized matrix within the calcium bodies begins. The apical surface of the anterior calcium body epithelium enlarges by forming numerous membrane folds and calcium transport across the anterior calcium body epithelium begins, as indicated by the intense folding of the apical epithelial surfaces and the presence of numerous electron dense granules in spaces between epithelial cells. Following the anterior ecdysis, degradation of mineralized matrix and intensive translocation of calcium take place in both pairs of calcium bodies. Within two days after the anterior ecdysis, mineral resorption from calcium bodies stops and the intermolt ultrastructure of calcium body epithelia is restored. At this point, the anterior calcium bodies are devoid of mineral content. In the posterior calcium bodies, only the bacteria-containing central matrix with apatite crystals remains and the glassy layer is completely degraded.

The continuously folded apical plasma membrane of the posterior calcium body epithelium indicates a constant high rate of transepithelial transport in the posterior calcium bodies. This is probably linked to the maintenance and functional regulation of the large bacterial population within the posterior calcium bodies. Epithelia with a highly folded or microvillous apical surface are also known from organs housing prokaryotic symbionts in other invertebrates, such as cephalopods [26] and insects [27].

The presence of calcium phosphate in bacteria-containing calcium bodies in *H. riparius* (this study) and *T. albus* [7] on one hand, and the absence of calcium phosphate in bacteria-free calcium bodies in *H. riparius* on the other, indicates that the function of bacteria is linked to calcium phosphate dynamics. However, bacteria might function in either its formation [28] or its dissolution [29], [30] and further investigation will be necessary to clarify their role.

Raman spectroscopy demonstrated calcite in concretions from the anterior calcium bodies. This result is unusual, since transient calcium deposits of crustaceans generally contain ACC [2]. It has been suggested that the amorphous nature of transient calcium deposits is functionally important, since ACC is highly soluble and thus readily resorbed when the need for calcium arises [1], [31]. Preparation methods used in this study should prevent the crystallization of ACC [31], [32] and amorphous minerals were well preserved in sternal deposits of *T. albus* (Fig. S2) and in the glassy layer of posterior calcium bodies. In crustaceans, ACC is generally stabilized by phosphate ions, proteins, and small organic molecules [2], [23], [33]. It is noteworthy that sternal deposits contain some phosphate and large amounts of organic material (Fig. S2), whereas no phosphate and comparatively small amounts of organic components were detected in the concretions within anterior calcium bodies of *H. riparius*. If the CaCO_3_ in anterior calcium bodies is amorphous *in vivo*, which is not excluded, it is possible that the heating during Raman spectroscopic measurement caused the crystallization of the samples. Whatever the *in vivo* condition, CaCO_3_ is readily resorbed from anterior calcium bodies after ecdysis.

The posterior calcium bodies in *H. riparius* are structurally and functionally similar to the calcium bodies of *T. albus* [7]. The continuously present crystalline mineral at the center of the posterior calcium bodies is apatite, as is the case in *T. albus*, whereas the transient glassy layer contains a mixture of calcium carbonate and calcium phosphate. Previously, energy dispersive X-ray spectrometry showed large amounts of calcium and phosphorus in the glassy layer in calcium bodies of *T. albus*; however, the Ca:P ratio was higher than would be expected for either apatite or ACP alone [7], which indicated that a mixture of minerals was present in this layer. In *H. riparius*, ACC and ACP form the glassy layer. If brushite is also present, as indicated by Raman spectroscopy, it should be readily resorbed from calcium bodies since its solubility [22] is comparable to that of ACC [34]. Unlike the glassy layer, apatite crystals in posterior calcium bodies are always present and we could not establish any changes to the bacteria-containing central matrix during the molt cycle. The same was observed in *T. albus* [7]. The function of apatite in calcium bodies thus remains unknown.

Calcium phosphate has more rarely been reported in crustacean calcium deposits than calcium carbonate. It represents a small fraction of the minerals in sternal deposits of *Porcellio scaber* [35] and was also detected in the calcium concretions within the posterior midgut caeca of *Orchestia cavimana* [23]. Although the function of calcium phosphates in the posterior calcium bodies is not known at this point, it is possible that they represent a reservoir for phosphorus in addition to calcium. It has been shown that the tergal cuticle of terrestrial isopods contains measurable amounts of calcium phosphate [36], [37], [38], [39], predominantly in the proximal cuticular layers, where it supposedly stabilizes calcium carbonate in amorphous form [39]. Calcium phosphate in the glassy layer of the posterior calcium bodies could therefore provide a source of phosphate ions necessary for the mineralization of the exoskeleton.

If we assume that the etched appearance of the mineralized matrix within calcium bodies and the presence of electron dense granules in intercellular spaces are indicative of calcium resorption, there is evidently a delay between the onset of mineral resorption in the anterior and the posterior calcium bodies. This could be linked to the biphasic molt, but if we relate this sequence of events to the composition of the minerals that are resorbed in the two pairs of calcium bodies, we can conclude that only calcium carbonate is resorbed during intramolt stage, while both calcium carbonate and calcium phosphate are resorbed during postmolt stage, the resorption of calcium phosphate perhaps being linked to the phosphate-stabilized ACC mineralization of the endocuticle [39].

The ultrastructural features of the calcium body epithelium during mineral resorption are nearly identical to those reported for the anterior sternal epithelium during the resorption of sternal deposits in other oniscids [13]. The deeply folded apical plasma membrane, an extensive intercellular network between adjacent epithelial cells and electron dense granules forming within these spaces on membrane protrusions, observable in *H. riparius*, are also characteristics of the anterior sternal epithelium during resorption of the sternal CaCO_3_ deposits of terrestrial isopods [13], [40]. The electron dense extracellular granules were shown to contain Ca, N [40] and P [35] in the terrestrial isopod *Porcellio scaber*, which indicates that they function in transepithelial calcium transport in this species. Considering these ultrastructural similarities, it is likely that similar cellular processes enable the transepithelial transport of calcium in calcium bodies and in sternal deposits of isopods. Similar features were reported in the epithelium of the posterior midgut caeca during resorption of mineral concretions in the amphipod *Orchestia cavimana* [41] and the epidermis during resorption of cuticular calcium in the crab, *Holthuisana transversa* [42]. This indicates that different crustacean groups utilize a similar mechanism of transepithelial calcium transport. Interestingly, electron dense granules were not observed during mineral resorption from calcium bodies in *T. albus* [7], which might be the consequence of the generally slower metabolism in this subterranean species [43] and the consequently lower intensity of calcium transport.

*H. riparius* is the only known terrestrial isopod lacking sternal CaCO_3_ deposits apart from the tylid *Helleria brevicornis*, which was reported to store calcium in the hemolymph [8]. Sternal deposits are utilized by representatives of all major lineages of terrestrial isopods, including Tylidae [8], Ligiidae [44], [45], at least three members of Synocheta [8], [46], and all studied Crinocheta [45]. Despite many different views on oniscidean phylogeny [47], the absence of sternal deposits in *Hyloniscus* is likely the result of secondary reduction considering their presence in so many oniscidean lineages. It is possible that the anterior calcium bodies functionally replace sternal CaCO_3_ deposits in this species.

Complete reliance on internal calcium deposits may be advantageous from the perspective of the timing of mineral deposition and resorption processes. CaCO_3_ deposits in the ecdysial space can only start forming after the onset of apolysis. By contrast, mineral can already be deposited in calcium bodies before this point in the molting cycle, and our results demonstrate that this is the case in anterior calcium bodies of *H. riparius*. Whether the matrix formed at this point is mineralized with dietary calcium or with calcium resorbed from the exoskeleton remains to be established. Furthermore, sternal deposits are necessarily degraded during a relatively short period in intramolt stage if the animal is to avoid losing them with the anterior exuviae. The time available for the resorption of sternal deposits is thus limited to this narrow timeframe, which is impractical, as much of the endocuticle, in particular of the anterior body-half, is synthesized after the anterior ecdysis and calcium is still required for its mineralization in postmolt stage [4]. Calcium from an internal deposit, on the other hand, can be resorbed after ecdysis and we demonstrated that resorption of minerals from both pairs of calcium bodies in *H. riparius* is most intensive during the postmolt stage. Furthermore, the chemical environment within an internal epithelial compartment may be more efficiently regulated compared to the ecdysial space, making the utilization of calcium bodies energetically favorable.

We could not establish any luminal connections between the calcium bodies and the digestive system. However, they are located close to digestive organs and the basal lamina of the calcium body epithelium is often in direct contact with that of the hepatopancreas. It is therefore possible that ions are transported almost directly between the calcium bodies and components of the digestive system despite the absence of a luminal connection.

Our results demonstrate that new envelopes are formed by the calcium body epithelium before each molt. Since calcium bodies are closed compartments, this should lead to accumulation of old envelopes within the calcium body lumen unless they are degraded. Since numerous laminae are observable within the lumen of both pairs of calcium bodies, and the structure and thickness of these laminae are identical to the electron lucent sublayer of the calcium body envelopes, it is likely that they are indeed accumulated remnants of old envelopes, secreted during previous molts. The mineralized matrix formed in the premolt stage is closely associated with the old envelopes. It is therefore possible that the old envelopes function in the initiation of mineral deposition.

## Supporting Information

Figure S1 **Absence of sternal deposits in** ***Hyloniscus riparius*** **.** (A) Ventral view of anterior pereonites in late premolt stage. No sternal deposits are visible on the anterior sternites (st). (B) Ventral view of anterior pereonites in intramolt stage. No sternal deposits are visible. (C) The anterior sternal epithelium in intramolt stage. The epithelium (e) lacks pronounced apical membrane invaginations, epithelial cells are in close contact with each other and no electron dense granules are visible in intercellular spaces. ct – cephalothorax, es – ecdysial space, j – cell junction, nc – new cuticle.
(TIF)

Figure S2 **Raman spectrum of sternal deposits in** ***Titanethes albus*** **.** Positions of the most prominent peaks are given in cm^−1^. Asterisk denotes spike. The peaks at 957 cm^−1^, 1081 cm^−1^, and 2935 cm^−1^ are contributed by calcium phosphate, calcium carbonate, and organic components, respectively.
(TIF)
