# Supplementary figures and images for: Mineral Deposition in Bacteria-Filled and Bacteria-Free Calcium Bodies in the Crustacean Hyloniscus riparius (Isopoda: Oniscidea)
Source: PLoS One. 2013 Mar 12;8(3):e58968. doi: 10.1371/journal.pone.0058968 (PMC3595210; doi:10.1371/journal.pone.0058968)

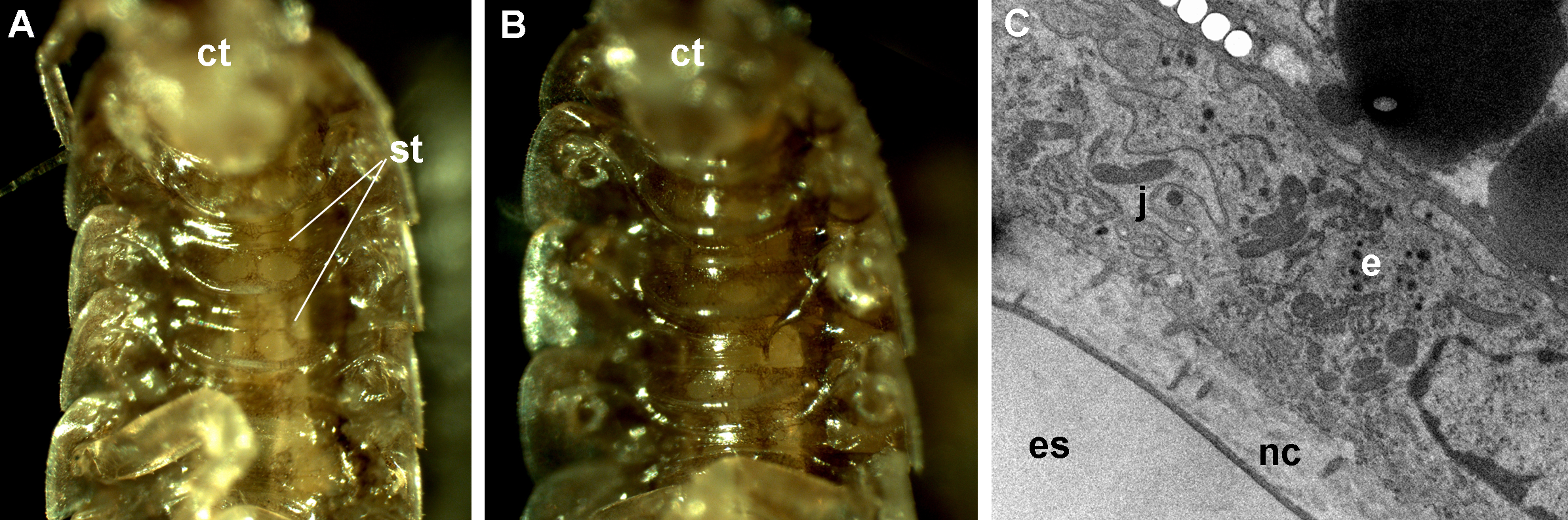

Supplement: Figure S1 — Absence of sternal deposits in Hyloniscus riparius . (A) Ventral view of anterior pereonites in late premolt stage. No sternal deposits are visible on the anterior sternites (st). (B) Ventral view of anterior pereonites in intramolt stage. No sternal deposits are visible. (C) The anterior sternal epithelium in intramolt stage. The epithelium (e) lacks pronounced apical membrane invaginations, epithelial cells are in close contact with each other and no electron dense granules are visible in intercellular spaces. ct – cephalothorax, es – ecdysial space, j – cell junction, nc – new cuticle. (TIF) [file pone.0058968.s001.tif]

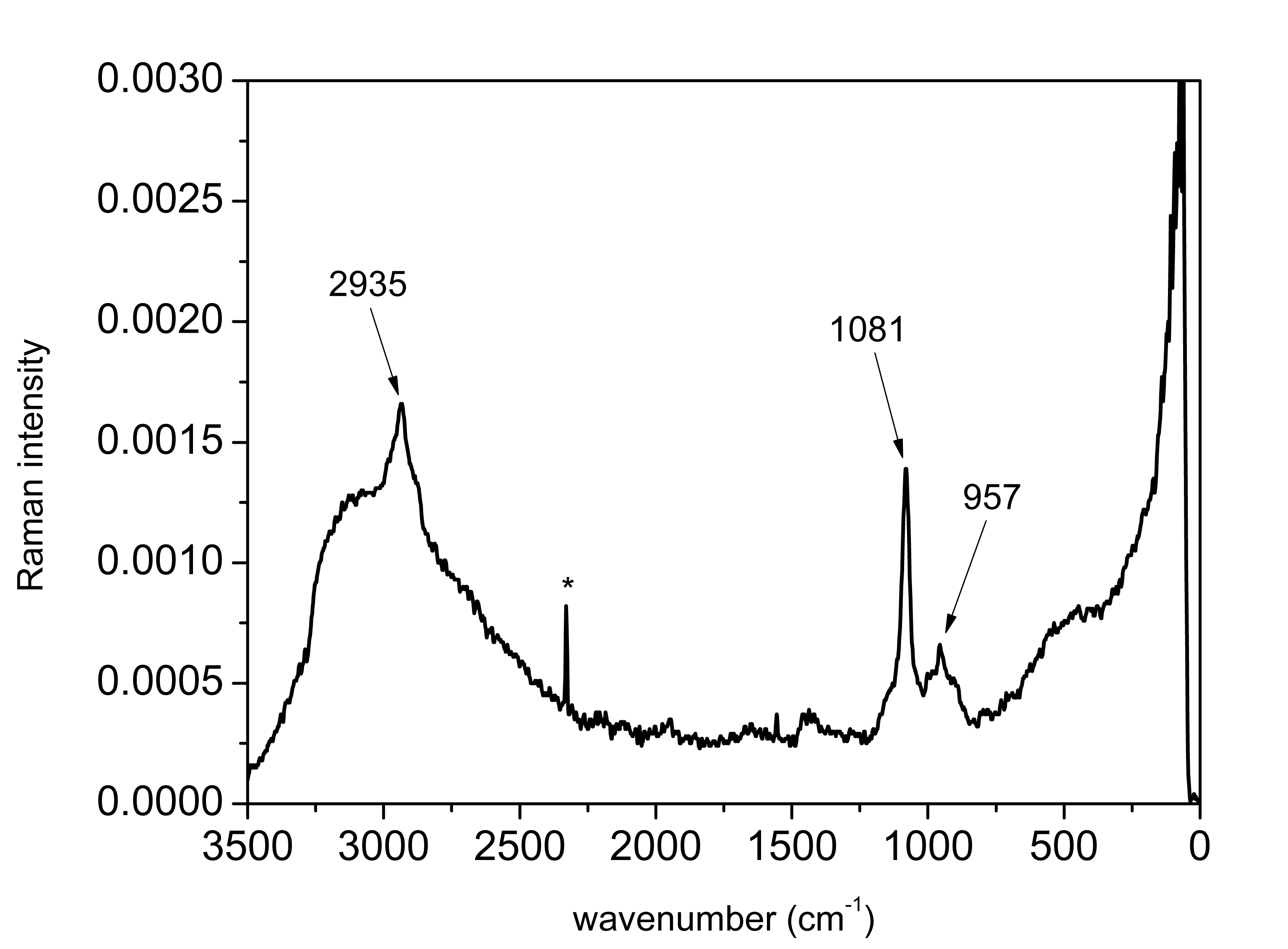

Supplement: Figure S2 — Raman spectrum of sternal deposits in Titanethes albus . Positions of the most prominent peaks are given in cm−1. Asterisk denotes spike. The peaks at 957 cm−1, 1081 cm−1, and 2935 cm−1 are contributed by calcium phosphate, calcium carbonate, and organic components, respectively. (TIF) [file pone.0058968.s002.tif]
